# Phylogenetic relationships of cone snails endemic to Cabo Verde based on mitochondrial genomes

**DOI:** 10.1186/s12862-017-1069-x

**Published:** 2017-11-25

**Authors:** Samuel Abalde, Manuel J. Tenorio, Carlos M. L. Afonso, Juan E. Uribe, Ana M. Echeverry, Rafael Zardoya

**Affiliations:** 1 Museo Nacional de Ciencias Naturales (MNCN-CSIC), José Gutiérrez Abascal 2, 28006 Madrid, Spain; 2 Departamento CMIM y Q. Inorgánica-INBIO, Facultad de Ciencias, Universidad de Cádiz, 11510 Puerto Real, Cádiz, Spain; 3 Centre of Marine Sciences (CCMAR), Universidade do Algarve, Campus de Gambelas, 8005 - 139 Faro, Portugal

**Keywords:** Mitochondrial genomes, *Africonus*, *Trovaoconus*, *Kalloconus*

## Abstract

**Background:**

Due to their great species and ecological diversity as well as their capacity to produce hundreds of different toxins, cone snails are of interest to evolutionary biologists, pharmacologists and amateur naturalists alike. Taxonomic identification of cone snails still relies mostly on the shape, color, and banding patterns of the shell. However, these phenotypic traits are prone to homoplasy. Therefore, the consistent use of genetic data for species delimitation and phylogenetic inference in this apparently hyperdiverse group is largely wanting. Here, we reconstruct the phylogeny of the cones endemic to Cabo Verde archipelago, a well-known radiation of the group, using mitochondrial (mt) genomes.

**Results:**

The reconstructed phylogeny grouped the analyzed species into two main clades, one including *Kalloconus* from West Africa sister to *Trovaoconus* from Cabo Verde and the other with a paraphyletic *Lautoconus* due to the sister group relationship of *Africonus* from Cabo Verde and *Lautoconus ventricosus* from Mediterranean Sea and neighboring Atlantic Ocean to the exclusion of *Lautoconus* endemic to Senegal (plus *Lautoconus guanche* from Mauritania, Morocco, and Canary Islands). Within *Trovaoconus*, up to three main lineages could be distinguished. The clade of *Africonus* included four main lineages (named I to IV), each further subdivided into two monophyletic groups. The reconstructed phylogeny allowed inferring the evolution of the radula in the studied lineages as well as biogeographic patterns. The number of cone species endemic to Cabo Verde was revised under the light of sequence divergence data and the inferred phylogenetic relationships.

**Conclusions:**

The sequence divergence between continental members of the genus *Kalloconus* and island endemics ascribed to the genus *Trovaoconus* is low, prompting for synonymization of the latter. The genus *Lautoconus* is paraphyletic. *Lautoconus ventricosus* is the closest living sister group of genus *Africonus*. Diversification of *Africonus* was in allopatry due to the direct development nature of their larvae and mainly triggered by eustatic sea level changes during the Miocene-Pliocene. Our study confirms the diversity of cone endemic to Cabo Verde but significantly reduces the number of valid species. Applying a sequence divergence threshold, the number of valid species within the sampled *Africonus* is reduced to half.

**Electronic supplementary material:**

The online version of this article (10.1186/s12862-017-1069-x) contains supplementary material, which is available to authorized users.

## Background

The cone snails (Conidae, Gastropoda) endemic to the archipelago of Cabo Verde in West Africa represent one of the few textbook examples of a well-documented insular species radiation involving marine organisms [1–3]. Cone snails, which are found in tropical and subtropical marine waters throughout the world, show a hotspot of species diversity in the Cabo Verde archipelago with up to 95 endemic species (roughly 10% of cone species diversity worldwide) narrowly confined to about 4000 km^2^ [4]. As in other parts of the world, cone snails endemic to Cabo Verde constitute a key component of the intertidal and subtidal ecosystems associated to rocky shores, coral reefs, and sandy bottoms. All cones endemic to Cabo Verde feed on marine annelid worms [1] and use a sophisticated venom apparatus (including a venom gland that produces conotoxins and a specialized harpoon-like radular tooth) to capture their preys [5]. Another interesting biological feature common to all these endemic species is that they have direct development. Their larvae lack a pelagic stage, and thus show a considerably reduced dispersal capacity [1]. Survival rate is higher for this type of larvae since they are less likely to be eaten by predators and are not dependent on plankton for feeding (i.e, non-planktotrophic).

The origin and evolutionary history of cones endemic to Cabo Verde has been the subject of several recent phylogenetic studies [1, 2, 6, 7]. Molecular phylogenies demonstrated that two different ancestors reached the archipelago independently and subsequently diversified following recurrent biogeographic patterns [1, 2, 7]. The existence of two clades led to the classification of cone species endemic to Cabo Verde into two genera, *Africonus* and *Trovaoconus* [8]. The question of which species are the closest living sister groups to *Africonus* and *Trovaoconus* remains open [1, 2]. According to a previous study, the ancestor of *Africonus* colonized the archipelago in the Miocene, about 16.5 million years ago (mya; [1]), and spread to all islands (except Fogo, the youngest, with steep slopes in the coast and ongoing volcanic activity). Most (95%) of the currently described species endemic to Cabo Verde belong to *Africonus*, and are normally referred to as restricted to a single island and in some cases even to single bays within an island [3]. The ancestor of *Trovaoconus* arrived at Cabo Verde archipelago in the Pliocene, about 4.6 mya, and diversified only in four islands (Sal, Boa Vista, Maio, and possibly Santiago), which are the closest to the continent [1]. These cones are significantly larger in size than those belonging to *Africonus* and show wider distributions extending in some cases to more than one island. It has been hypothesized that diversification within each genus was in allopatry and followed recurrent eustatic sea level changes during the Neogene that intermittently connected and disconnected the islands [1, 7]. However, sea level fluctuations alone do not fully explain the extraordinary diversity of cones in Cape Verde since nearby archipelagos in the Macaronesia biogeographic region such as the Canary Islands subjected to similar trends since the Miocene do not have endemic cone species [6]. A larger distance to the mainland, which enhances isolation and restricts gene flow combined with a higher mean sea surface temperature and the presence of more suitable habitats may have promoted a significant increase in diversification rates in the Cabo Verde archipelago [6].

**Fig. 1:**
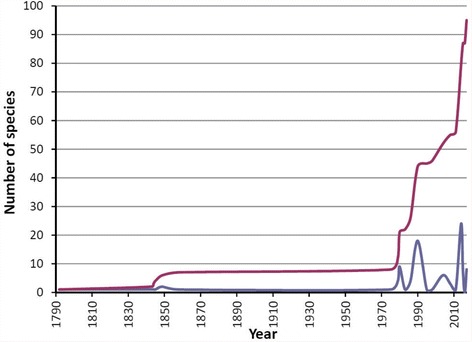
Number (blue) and accumulated number (red) of cone species described for the Cabo Verde archipelago per year

The rate of description of new cone species endemic to Cabo Verde has accelerated more than expected during the last years (Fig. 1). After the early descriptions in the eighteenth and nineteenth centuries based on samples brought to Europe by naturalists [9], the main contribution to the cataloguing of cone species endemic to Cabo Verde was due to the work of Emilio Rolán [10], who drew attention to this singular radiation. Hence, around year 2000, there were about 50 species recognized [11] and remarkably this number has almost doubled in the last 2-3 years [12–19]. However, it is important to note that many of the recent species diagnoses in cones are mainly based on the shape, color, and banding patterns of the shell. These phenotypic characters are highly variable at the population level and prone to local adaptation and convergence, making species assignment problematic and sometimes, misleading [7]. In many cases, distinguishing whether different shell morphotypes of cone snails represent valid species or ecotypes of the same species is challenging [20]. Therefore, determination of genetic variation and inference of phylogenetic relationships based on DNA sequence data are timely as part of a multidisciplinary approach [21] to identify and delimit species and to understand evolutionary processes underlying diversification within cones, in general, and within those endemic to Cabo Verde, in particular.

Here, we used nearly complete mitochondrial (mt) genomes, which have proven to successfully reconstruct robust phylogenies of Conidae [22] and of particular groups such as the cones endemic to Senegal [23]. In this study, we sequenced the nearly complete mt genomes of 88 individuals representing different populations and species of *Africonus* and *Trovaoconus* endemic to Cabo Verde. We aimed to: (1) reconstruct a highly resolved phylogeny of cones endemic to Cabo Verde; (2) determine the closest living sister groups of *Africonus* and *Trovaoconus*; (3) date major cladogenetic events and analyze biogeographical patterns; (4) study radular tooth evolution within the two genera; and (5) provide a first genetic hypothesis of species delimitation in the radiation of Cabo Verde endemic cones.

## Results

### Sequencing, assembly, and genome organization

The nucleotide sequences of the near-complete mt genomes of 75 specimens of *Africonus*, 13 specimens of *Trovaoconus*, and one specimen of *Lautoconus ventricosus* were determined (Table 1). These mt genomes lacked the *trnF* gene, the control region, and the start of the *cox3* gene because the corresponding fragment was not PCR amplified. The number of reads, mean coverage, and length of each mt genome are provided in Table 1. The mt genomes of *Africonus boavistensis* and *Africonus denizi* received the minimum (42,021) and maximum (906,765) number of reads, respectively. The same samples received the minimum (412×) and maximum (8,885×) mean coverage, respectively (Table 1). All sequenced mt genomes encode for 13 protein-coding, 2 rRNA and 21 tRNA genes (but note that the *trnF* gene could not be determined; see above). They all share the same genome organization: the major strand encodes all genes, except those forming the cluster MYCWQGE (*trnM*, *trnY*, *trnC*, *trnW*, *trnQ*, *trnG*, *trnE*) and the *trnT* gene.

**Table 1:** Mitochondrial (mt) genomes analyzed in this study

New mt genomes										
ID CV	Initial species identification	Location	Coordinates	Coverage		Length (bp)	GenBank Acc. No	Voucher DNA (MNCN/ADN)	Voucher shell (MNCN 15.05/)	New species proposed^a^
				n° reads	mean depth					
1020	*Africonus antoniaensis*	Água Doce, Boa Vista, Cabo Verde	16°12'29"N, 22°44'7"W	151104	1476.8	15332	MF491587	95072	79889	*—*
0885	*Africonus antoniomonteiroi*	Pedra Lume, Sal, Cabo Verde	16°45'44"N, 22°53'2"W	232069	2273.4	15328	MF491578	95063	79794	*—*
0927	*Africonus bernardinoi*	Pedra Lume, Sal, Cabo Verde	16°45'44"N, 22°53'2"W	59799	583.3	15328	MF491582	95067	79835	*Africonus cuneolus*
0520	*Africonus boavistensis*	Baía do Ervatão (North), Boa Vista, Cabo Verde	16°12'3"N, 22°54'43"W	42021	412.8	15217	MF491563	95045	80413	*—*
1135	*Africonus cabraloi*	Estancinha, Boa Vista, Cabo Verde	16°13'12"N, 22°55'9"W	74446	730.4	15329	MF491598	95083	80004	*Africonus crotchii*
0895	*Africonus cagarralensis*	Pedra Lume, Sal, Cabo Verde	16°45'44"N, 22°53'2"W	161290	1367.2	15320	MF491579	95064	79804	*Africonus longilineus*
0173	*Africonus calhetae*	Praia da Soca, Maio, Cabo Verde	15°15'8"N, 23°13'4"W	55433	544.7	15242	MF491534	95016	78798	*—*
0920	*Africonus* cf. *anthonyi*	Ilhéus do Chano, Sal, Cabo Verde	16°41'37"N, 22°52'47"W	172336	1678.6	15315	MF491581	95066	79828	*Africonus cuneolus*
0162	*Africonus* cf. *claudiae*	Praia da Soca, Maio, Cabo Verde	15°15'8"N, 23°13'4"W	87407	858.6	15326	MF491533	95015	78787	*Africonus calhetae*
0465	*Africonus* cf. *delanoyae*	Ponta Antónia, Boa Vista, Cabo Verde	16°13'24"N, 22°46'59"W	382817	3736.1	15335	MF491559	95041	80409	*Africonus fuscoflavus*
0207	*Africonus* cf. *galeao*	Ponta Pipa, Maio, Cabo Verde	15°19'30"N, 23° 9'48"W	81447	797.3	15325	MF491536	95018	78832	*Africonus galeao*
0135	*Africonus* cf. *gonsaloi*	Praia Gonçalo, Maio, Cabo Verde	15°16'13"N, 23°6'15"W	148032	1455.5	15250	MF491529	95011	78760	*Africonus gonsaloi*
0380	*Africonus* cf. *miguelfiaderoi*	Jorrita, Baía da Gata, Boa Vista, Cabo Verde	16°12'9"N, 22°42'22"W	358342	3507.9	15328	MF491548	95030	80398	*Africonus vulcanus*
1400	*Africonus* cf. *miruchae*	Calhau, São Vicente, Cabo Verde	16°51'7"N, 24°51'59"W	523002	5104.9	15321	MF491601	95088	78562	*Africonus* sp. nov. 1
0223	*Africonus claudiae*	Ponta Pipa, Maio, Cabo Verde	15°19'30"N, 23° 9'48"W	148508	1434.5	15337	MF491537	95019	78848	*Africonus galeao*
0303	*Africonus condei*	Baía Grande, Derrubado, Boa Vista, Cabo Verde	16°13'31"N, 22°47'17"W	253863	2472.2	15248	MF491542	95024	80392	*Africonus crotchii*
0045	*Africonus crioulus*	Praia Santana, Maio, Cabo Verde	15°18'13"N, 23°11'49"W	255019	2502	15247	MF491521	95003	78670	*Africonus maioensis*
1075	*Africonus crotchii*	Morro de Areia, Boa Vista, Cabo Verde	16°5'24"N, 22°57'7"W	332385	3237.6	15329	MF491591	95076	79944	*—*
0803	*Africonus cuneolus*	Calheta Funda, Sal, Cabo Verde	16°39'6"N, 22°56'53"W	184181	1791.6	15329	MF491569	95053	79712	*—*
0936	*Africonus cuneolus*	Santa Maria, Sal, Cabo Verde	16°35'38"N, 22°53'36"W	80472	787.4	15328	MF491583	95068	79844	*—*
1420	*Africonus curralensis*	Praia de Palmo Tostão, Santa Luzia, Cabo Verde	16°45'19"N, 24°45'24"W	857123	8358.6	15329	MF491602	95089	78581	*—*
1017	*Africonus damioi*	Água Doce, Boa Vista, Cabo Verde	16°12'29"N, 22°44'7"W	76477	745.5	15326	MF491586	95071	79886	*Africonus roeckeli*
0405	*Africonus damottai*	Baía da Gata (center), Boa Vista, Cabo Verde	16°11'50"N, 22°42'32"W	315488	2914.3	15358	MF491551	95033	80401	*—*
1428	*Africonus decoratus*	Curral, Santa Luzia, Cabo Verde	16°46'23"N, 24°47'13"W	566822	5540.2	15326	MF491603	95090	78589	*—*
0370	*Africonus delanoyae*	Jorrita, Baía da Gata, Boa Vista, Cabo Verde	16°12'9"N, 22°42'22"W	158489	1543.7	15323	MF491547	95029	80397	*—*
1471	*Africonus denizi*	Praia Grande, São Vicente, Cabo Verde	16°51'40"N, 24°52'30"W	906765	8885.2	15326	MF491605	95092	78621	*—*
0315	*Africonus derrubado*	Baía Grande, Derrubado, Boa Vista, Cabo Verde	16°13'31"N, 22°47'17"W	214173	2089.2	15243	MF491543	95025	80393	*Africonus damottai*
0565	*Africonus diminutus*	Ilhéu de Sal Rei, Boa Vista, Cabo Verde	16°9'50"N, 22°55'31"W	840424	8204.1	15330	MF491566	95049	80416	*—*
1025	*Africonus docensis*	Água Doce, Boa Vista, Cabo Verde	16°12'29"N, 22°44'7"W	47313	464.8	15329	MF491588	95073	79894	*Africonus crotchii*
0385	*Africonus evorai*	Zebraca (near Ilhéu do Galeão), Boa Vista, Cabo Verde	16°12'6"N, 22°42'40"W	226416	2218	15243	MF491549	95031	80399	*Africonus crotchii*
0070	*Africonus fantasmalis*	Porto Cais, Maio, Cabo Verde	15°19'15"N, 23°11'10"W	97527	954.7	15330	MF491524	95006	78695	*Africonus fuscoflavus*
0835	*Africonus felitae*	Rabo de Junco, Sal, Cabo Verde	16°41'44"N, 22°58'35"W	344190	3343.9	15404	MF491573	95057	79744	*—*
1437	*Africonus fernandesi*	Porto Novo, Santo Antão, Cabo Verde	17°1'4"N, 25°3'22"W	742414	7244.2	15324	MF491604	95091	78598	*—*
0332	*Africonus fiadeiroi*	Derrubado (bay West), Boa Vista, Cabo Verde	16°13'22"N, 22°47'41"W	205910	2016.5	15243	MF491545	95027	80395	*Africonus crotchii*
0855	*Africonus fontonae*	Baía da Fontona, Sal, Cabo Verde	16°44'22"N, 22°58'46"W	156259	1523.9	15328	MF491575	95059	79764	*Africonus cuneolus*
0945	*Africonus fontonae*	Regona, Sal, Cabo Verde	16°48'5"N, 22°59'33"W	56310	549.8	15327	MF491584	95069	79853	*Africonus regonae*
0450	*Africonus fuscoflavus*	Derrubado (bay East), Boa Vista, Cabo Verde	16°13'331"N, 22°47'3"W	151904	1478.6	15331	MF491557	95039	80407	*—*
0052	*Africonus galeao*	Navio Quebrado, Terras Salgadas, Maio, Cabo Verde	15°18'54"N, 23°11'2"W	117940	1139.2	15326	MF491522	95004	78677	*—*
0134	*Africonus gonsaloi*	Praia Gonçalo, Maio, Cabo Verde	15°16'13"N, 23°6'15"W	188174	1835.9	15339	MF491528	95010	78759	*—*
1390	*Africonus grahami*	Calhau, São Vicente, Cabo Verde	16°51'7"N, 24°51'59"W	464704	4536.1	15325	MF491599	95086	78552	*—*
0140	*Africonus irregularis*	Porto Cais (North), Maio, Cabo Verde	15°19'45"N, 23°10'57"W	202254	1937.9	15321	MF491530	95012	78765	*Africonus maioensis*
0317	*Africonus irregularis*	Baía Grande, Derrubado, Boa Vista, Cabo Verde	16°13'31"N, 22°47'17"W	170523	1668.2	15331	MF491544	95026	80394	*Africonus maioensis*
0392	*Africonus irregularis*	Baía da Gata, Boa Vista, Cabo Verde	16°11'50"N, 22°42'32"W	252126	2454.3	15324	MF491550	95032	80400	*Africonus crotchii*
1084	*Africonus irregularis*	Morro de Areia, Boa Vista, Cabo Verde	16°5'24"N, 22°57'7"W	125264	1225.1	15330	MF491593	95078	79953	*Africonus crotchii*
1128	*Africonus irregularis*	Estancinha, Ponta do Sol, Boa Vista, Cabo Verde	16°13'12"N, 22°55'9"W	469101	4597.1	15313	MF491597	95082	79997	*Africonus crotchii*
0225	*Africonus isabelarum*	Ponta do Pau Seco, Maio, Cabo Verde	15°15'26"N, 23°13'16"W	247567	2431.7	15244	MF491538	95020	78850	*—*
0085	*Africonus josephinae*	Lage Branca, Maio, Cabo Verde	15°18'32"N, 23°8'17"W	224495	2204.6	15239	MF491525	95007	78710	*Africonus* sp. nov. 2
0555	*Africonus josephinae*	Ilhéu de Sal Rei, Boa Vista, Cabo Verde	16°9'50"N, 22°55'31"W	169723	1611.8	15330	MF491565	95048	80415	*—*
0830	*Africonus longilineus*	Serra Negra, Sal, Cabo Verde	16°38'17"N, 22°53'56"W	148726	1453	15316	MF491572	95056	79739	*—*
0847	*Africonus longilineus*	Rabo de Junco, Sal, Cabo Verde	16°41'44"N, 22°58'35"W	308057	3000.9	15333	MF491574	95058	79756	*Africonus miruchae*
0410	*Africonus luquei*	Praia Canto, Boa Vista, Cabo Verde	16°11'10"N, 22°42'28"W	83198	815.1	15244	MF491552	95034	80402	*Africonus delanoyae*
0064	*Africonus maioensis*	Porto Cais, Maio, Cabo Verde	15°19'15"N, 23°11'10"W	143797	1402.9	15327	MF491523	95005	78689	*—*
0510	*Africonus marckeppensi*	Ervatao Norte, Boa Vista, Cabo Verde	16°12'3"N, 22°54'43"W	254030	2480.3	15330	MF491562	95044	80412	*Africonus josephinae*
0102	*Africonus marcocastellazzii*	Lage Branca, Maio, Cabo Verde	15°18'32"N, 23°8'17"W	80254	783.1	15326	MF491527	95009	78727	*Africonus maioensis*
0870	*Africonus melissae*	Baía da Parda, Sal, Cabo Verde	16°45'7"N, 22°53'56"W	195531	1907.9	15328	MF491577	95061	79779	*Africonus longilineus*
0455	*Africonus messiasi*	Derrubado (bay East), Boa Vista, Cabo Verde	16°13'33"N, 22°47'3"W	250171	2447.7	15260	MF491558	95040	80408	*Africonus fuscoflavus*
0426	*Africonus miguelfiaderoi*	Praia Canto, Boa Vista, Cabo Verde	16°11'10"N, 22°42'28"W	130100	1270.5	15328	MF491554	95036	80404	*Africonus vulcanus*
0905	*Africonus mordeirae*	Baía do Roucamento, Sal, Cabo Verde	16°41'20"N, 22°56'24"W	99337	971.4	15241	MF491580	95065	79814	*Africonus cuneolus*
1091	*Africonus morroensis*	Morro de Areia, Boa Vista, Cabo Verde	16°5'24"N, 22°57'7"W	172039	1515.1	15337	MF491594	95079	79960	*Africonus diminutus*
1395	*Africonus navarroi*	Calhau, São Vicente, Cabo Verde	16°51'7"N, 24°51'59"W	665250	6509.2	15331	MF491600	95087	78557	*—*
0250	*Africonus nelsontiagoi*	Tarrafal, Santiago, Cabo Verde	15°16'50"N, 23°45'15"W	173873	1687.2	15339	MF491541	95023	78875	*Africonus verdensis*
0820	*Africonus pseudocuneolus*	Serra Negra, Sal, Cabo Verde	16°38'17"N, 22°53'56"W	131838	1288.7	15337	MF491571	95055	79729	*Africonus cuneolus*
0036	*Africonus raulsilvai*	Praia da Soca, Maio, Cabo Verde	15°15'8"N, 23°13'4"W	345872	1699.6	15534	MF491520	95002	78661	*—*
0865	*Africonus regonae*	Baía da Fontona, Sal, Cabo Verde	16°44'22"N, 22°58'46"W	246041	2411	15328	MF491576	95060	79774	*—*
0950	*Africonus regonae*	Regona, Sal, Cabo Verde	16°48'5"N, 22°59'33"W	88673	864.1	15337	MF491585	95070	79858	*—*
0586	*Africonus roeckeli*	Praia Canto, Boa Vista, Cabo Verde	16°11'10"N, 22°42'28"W	141600	1385.7	15320	MF491567	95050	80417	*—*
0549	*Africonus salreiensis*	Ilhéu de Sal Rei, Boa Vista, Cabo Verde	16°9'50"N, 22°55'31"W	349070	3402.9	15331	MF491564	95047	80414	*Africonus crotchii*
0810	*Africonus serranegrae*	Serra Negra, Sal, Cabo Verde	16°38'17"N, 22°53'56"W	182124	1777.6	15335	MF491570	95054	79719	*Africonus cuneolus*
1078	*Africonus silviae*	Morro de Areia, Boa Vista, Cabo Verde	16°5'24"N, 22°57'7"W	293568	2876.1	15336	MF491592	95077	79947	*Africonus fuscoflavus*
0445	*Africonus swinneni*	Porto Ferreira, Boa Vista, Cabo Verde	16°7'45"N, 22°40'17"W	221672	2169.1	15244	MF491556	95038	80406	*Africonus delanoyae*
1125	*Africonus teodorae*	Estancinha, Ponta do Sol, Boa Vista, Cabo Verde	16°13'12"N, 22°55'9"W	326654	3185.5	15334	MF491596	95081	79994	*Africonus crotchii*
1035	*Africonus umbelinae*	Espingueira, Boa Vista, Cabo Verde	16°12'55"N, 22°47'49"W	110526	1085.5	15333	MF491589	95074	79904	*Africonus damottai*
0240	*Africonus verdensis*	Tarrafal, Santiago, Cabo Verde	15°16'50"N, 23°45'15"W	53400	519.8	15339	MF491540	95022	78865	*—*
0435	*Africonus vulcanus*	Porto Ferreira, Boa Vista, Cabo Verde	16°7'45"N, 22°40'17"W	284766	2787.4	15242	MF491555	95037	80405	*—*
1110	*Africonus zinhoi*	Curral Velho, Boa Vista, Cabo Verde	15°58'4"N, 22°47'42"W	388828	3785.5	15331	MF491595	95080	79979	*Africonus maioensis*
7036	*Trovaoconus atlanticoselvagem*	Baixo João Valente, Cabo Verde	15°44′27″N, 23°5′26″W	95264	933.8	15352	MF491606	7036	*—*	*Kalloconus trochulus*
0616	*Trovaoconus* cf. *ateralbus*	Serra Negra, Sal, Cabo Verde	16°38'17"N, 22°53'56"W	87550	853.7	15344	MF491568	95052	79664	*Kalloconus* sp. nov. 1
0010	*Trovaoconus pseudonivifer*	Ponta do Pau Seco, Maio, Cabo Verde	15°15'26"N, 23°13'16"W	56486	555.5	15351	MF491519	95000	78635	*Kalloconus trochulus*
0094	*Trovaoconus pseudonivifer*	Lage Branca, Maio, Cabo Verde	15°18'32"N, 23°8'17"W	454415	4429.8	15351	MF491526	95008	78719	*Kalloconus trochulus*
0154	*Trovaoconus pseudonivifer*	Porto Cais (north), Maio, Cabo Verde	15°19'45"N, 23°10'57"W	199736	1954.2	15352	MF491532	95014	78779	*Kalloconus trochulus*
0420	*Trovaoconus pseudonivifer*	Praia Canto, Boa Vista, Cabo Verde	16°11'10"N, 22°42'28"W	111182	1085	15347	MF491553	95035	80403	*Kalloconus pseudonivifer*
0500	*Trovaoconus trochulus*	Baía do Ervatão (North), Boa Vista, Cabo Verde	16°12'3"N, 22°54'43"W	223915	2177.2	15351	MF491561	95043	80411	*Kalloconus trochulus*
0149	*Trovaoconus venulatus*	Lage Branca, Maio, Cabo Verde	15°18'32"N, 23°8'17"W	105732	1014.9	15276	MF491531	95013	78774	*Kalloconus venulatus*
0187	*Trovaoconus venulatus*	Praia Real, Maio, Cabo Verde	15°19'45"N, 23°10'40"W	144651	1415	15330	MF491535	95017	78812	*Kalloconus venulatus*
0234	*Trovaoconus venulatus*	Ponta do Pau Seco, Maio, Cabo Verde	15°15'26"N, 23°13'17"W	67867	661.5	15320	MF491539	95021	78859	*Kalloconus venulatus*
0347	*Trovaoconus venulatus*	Derrubado (bay West), Boa Vista, Cabo Verde	16°13'22"N, 22°47'41"W	48636	475.9	15326	MF491546	95028	80396	*Kalloconus venulatus*
0475	*Trovaoconus venulatus*	Ponta Antónia, Boa Vista, Cabo Verde	16°13'24"N, 22°46'59"W	409041	3966.1	15340	MF491560	95042	80410	*Kalloconus venulatus*
1038	*Trovaoconus venulatus*	Praia Canto, Boa Vista, Cabo Verde	16°11'10"N, 22°42'28"W	143644	2403.8	15336	MF491590	95075	79907	*Kalloconus venulatus*
IB001	*Lautoconus ventricosus*	Estani des Peix, Formentera, Balearic Islands, Spain	38°43'49"N, 1°24'42"E	86290	842.5	15341	MF491607	95094	80426	*Lautoconus* sp. nov 1
GenBank mt genomes										
ID	Species	Location	Coordinates	Reference		Length (bp)	GenBank Acc. No	Voucher (MNCN/ADN)	Voucher shell (MNCN 15.05/)	New species proposed^a^
6990	*Africonus borgesi*	Porto Ferreira, Boa Vista, Cabo Verde	16°7'45"N, 22°40'170"W	Cunha et al., (2009)		15536	NC_013243	6990	*—*	*—*
0025	*Africonus infinitus*	Ponta do Pau Seco, Maio, Cabo Verde	15°15'26"N, 23°13'17"W	Abalde et al., (in prep.)		15522	KY864967	95001	78650	*—*
0875	*Africonus miruchae*	Terrinha Fina, Palhona, Sal, Cabo Verde	16°49'12"N, 22°59'12"W	Abalde et al., (in prep.)		15336	KY864971	95062	79784	*—*
0534	*Trovaoconus pseudonivifer*	Estancinha, Ponta do Sol, Boa Vista, Cabo Verde	16°13'12"N, 22°55'9"W	Abalde et al., (in prep.)		15351	KY864969	95046	80418	*Kalloconus trochulus*
0550	*Trovaoconus venulatus*	Ilhéu de Sal Rei, Boa Vista, Cabo Verde	16°9'56"N, 22°55'23"W	Uribe et al., (2017)		15524	KX263250	86741	80419	*Kalloconus venulatus*
0601	*Trovaoconus ateralbus*	Calheta Funda, Sal, Cabo Verde	16°39'6"N, 22°56'53"W	Abalde et al., (in prep.)		15327	KY864970	95051	79649	*Kalloconus ateralbus*
1375	*Kalloconus* cf. *byssinus*	North Senegal	unknown	Abalde et al., (in prep.)		15348	KY864973	95085	78536	*Kalloconus pulcher*
1253	*Kalloconus pulcher*	Les Almadies, Dakar, Senegal	14°44'40"N, 17°31'442"W	Abalde et al., (in prep.)		15332	KY864972	95084	78414	*Kalloconus pulcher*
1343	*Lautoconus belairensis*	Terrou-Bi. Dakar, Senegal	14°40'29"N, 17°28'12"W	Abalde et al., (2017)		15321	KY801849	91293	78504	*Gen. nov. belairensis*
1338	*Lautoconus bruguieresi*	Île de Gorée, Dakar, Senegal	14°40'16"N, 17°23'58"W	Abalde et al., (2017)		15340	KY801851	91291	78499	*Gen. nov. bruguieresi*
1296	*Lautoconus cloveri*	Ndayane, Senegal	14°33'45"N, 17°7'34"W	Abalde et al., (2017)		15323	KY801859	91283	78457	*Gen. nov. cloveri*
CG13	*Lautoconus guanche*	Lanzarote, Canary Islands, Spain	28°57'16"N, 13°34'22"W	Abalde et al., (2017)		15506	KY801847	91295	*—*	*Gen. nov. guanche*
1266	*Lautoconus hybridus*	NGor, Dakar, Senegal	14°45'67"N, 17°30'36.33"W	Abalde et al., (2017)		15507	KY801863	91279	78427	*Gen. nov. hybridus*
1278	*Lautoconus mercator*	NGor, Dakar, Senegal	14°45'6"N, 17°30'36"W	Abalde et al., (2017)		15329	KY801862	91280	78439	*Gen. nov. mercator*
CV13	*Lautoconus ventricosus*	Ria Formosa, Faro, Portugal	36°58'0"N, 7°53'2"W	Uribe et al., (2017)		15534	KX263251	86742	*—*	*—*
CVERM1	*Chelyconus ermineus*	Praia Gonçalo, Maio, Cabo Verde	15°16'13"N, 23°6'15"W	Abalde et al., (in prep.)		15365	KY864977	95095	78876	*—*

^a^hyphen indicates that original species name is maintained and considered valid

### Phylogenetic relationships and sequence divergences between clades

**Fig. 2:**
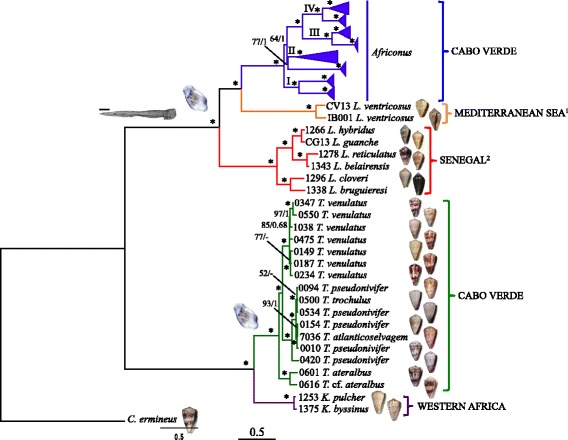
Closest sister groups of cone snails endemic to Cabo Verde based on mt genomes (concatenated protein coding plus rRNA genes analyzed at the nucleotide level). The reconstructed ML tree using *C. ermineus* as outgroup is shown. Number of specimen, initial species assignment, and a ventral picture of the shell are provided. Numbers at nodes are statistical support values for ML (bootstrap proportions)/BI (posterior probabilities). Scale bar indicates substitutions/site. Two major clades are recovered: the first one includes *Kalloconus* from Western Africa (purple) and *Trovaoconus* from Cabo Verde (green) whereas the second one includes *Lautoconus* from the Mediterranean Sea (and neighboring Atlantic Ocean^1^; orange), *Lautoconus* from Senegal (and *L. guanche* from Canary Islands^2^; red), and *Africonus* (blue) from Cabo Verde. Lineages within *Africonus* are expanded in Fig. 3. The “robust” type of radular tooth is shown as the ancestral character state for the second clade (scale bar equals 0.1 mm; see diversity of radular teeth of the first clade in Additional file 1). The two transitions from planktonic to non-planktonic larvae are indicated by a sac of eggs

**Fig. 3:**
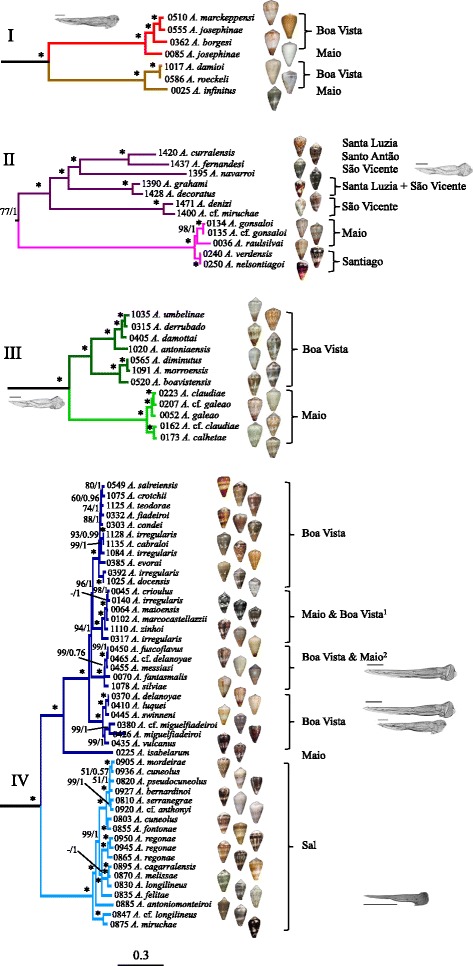
Phylogeny of *Africonus* based on mt genomes (concatenated protein coding plus rRNA genes analyzed at the nucleotide level). Number of specimen, initial species assignment, island, and a ventral picture of the shell are provided. Numbers at nodes are statistical support values for ML (bootstrap proportions)/BI (posterior probabilities). Hyphen indicates a bootstrap value below 50%. Scale bar indicates substitutions/site. Four major lineages (I-IV) are recovered and indicated with different colors. All *Africonus* have the “robust” type of radular tooth except when indicated (scale bar equals 0.1 mm). ^1^All taxa endemic to Maio except *A. irregularis* endemic to Boa Vista. ^2^All taxa endemic to Boa Vista except *A. fantamalis* endemic to Maio

Phylogenetic relationships of cones endemic to Cabo Verde were reconstructed based on the nucleotide sequences of the concatenated 13 mt protein-coding and two rRNA genes using probabilistic methods and *Chelyconus ermineus* as outgroup. The final matrix was 13,572 positions in length. According to the AIC, the best partition scheme for the protein-coding genes was the one combining all these genes but analyzing each codon position separately. The best substitution model for each of the three codon positions was GTR + I + G. For the rRNA genes, the best scheme had both genes combined under the GTR + I + G model. Both, ML (−*lnL* **=** 75,600.18) and BI (*−lnL* = 76,002.71 for run 1; *−lnL* = 76,288.44 for run 2) arrived at almost identical topology (Figs. 2 and 3). Most nodes received high statistical support and differences in topology were restricted exclusively to three relatively shallow nodes that had low support in ML and were unresolved in BI. Two of these nodes involved almost identical sequences and corresponded to *Africonus bernardinoi*/ *Africonus pseudocuneolus* and *Africonus teodorae*/ *Africonus fiadeiroi*, respectively. The third unresolved node corresponded to a trichotomy involving *Africonus felitae*, *Africonus regonae* and *Africonus longilineus*/ *Africonus cagarralensis*/ *Africonus melissae*.

The reconstructed phylogeny (Fig. 2) grouped the analyzed species into two main clades, one including *Kalloconus* from mainland West Africa sister to *Trovaoconus* from Cabo Verde and the other having paraphyletic *Lautoconus* due to the sister group relationship of *Africonus* from Cabo Verde and *Lautoconus ventricosus* from Mediterranean Sea and neighboring Atlantic Ocean to the exclusion of *Lautoconus* endemic to Senegal (plus *Lautoconus guanche* from Mauritania, Morocco, and Canary Islands). Within *Trovaoconus*, up to three main lineages could be distinguished (Fig. 2). The first one included two specimens from Sal initially identified as *Trovaoconus ateralbus*, which were sister to a clade including one lineage with specimens from Maio and Boa Vista identified as *Trovaoconus venulatus* and another lineage having mostly specimens of *Trovaoconus pseudonivifer* from Maio and Boa Vista but also one specimen of *Trovaoconus trochulus* from Boa Vista and one of *Trovaoconus atlanticoselvagem* from Baixo João Valente (Fig. 2).

The clade of *Africonus* from Cabo Verde included four main lineages (named I to IV), each further subdivided into two monophyletic groups (Figs. 2 and 3). Lineage I was the sister group of the remaining *Africonus* and its two lineages had each species from Maio sister to species from Boa Vista (Fig. 3a). Lineage II included species from Santiago and Maio sister to species endemic to the westernmost islands (Santo Antão, São Vicente and Santa Luzia). These latter species could be grouped into three main lineages, one containing species endemic to São Vicente, another containing species distributed both in Santa Luzia and São Vicente, and the third one including species from the three islands (Fig. 3a). Lineage III included species from Maio sister to species from Boa Vista (Fig. 3a). Lineage IV contained specimens representing most of the described species of *Africonus*. One monophyletic group included species endemic to Sal whereas the other clade included *Africonus isabelarum* from Maio as sister to four lineages, two containing exclusively species from Boa Vista, one having species from Maio sister to *Africonus irregularis* from Boa Vista, and one having species from Boa Vista and *Africonus fantasmalis* from Maio (Fig. 3b).

Pairwise uncorrected sequence divergences were estimated based on the alignment including the nucleotide sequences of the 13 mt protein-coding and two rRNA genes. Pairwise uncorrected sequence divergences between *C. ermineus* and ingroup taxa averaged 18%. The average pairwise uncorrected sequence divergence between the two main ingroup clades (genera *Kalloconus* + *Trovaoconus* versus genera *Lautoconus* + *Africonus*) was 16%. Pairwise uncorrected sequence divergences between *Lautoconus* endemic to Senegal (plus *L. guanche*) and *L. ventricosus* plus *Africonus* averaged 11%. The average pairwise uncorrected sequence divergence between the sister groups *L. ventricosus* and *Africonus* was 10% whereas between *Kalloconus* and *Trovaoconus,* it was 5%. The pairwise uncorrected sequence divergences between the four main lineages within *Africonus* averaged 6%. The corresponding values for the pairwise divergences between the two major clades defined within each of the lineages I-IV were 4%, 6%, 3%, and 3%, respectively. Pairwise uncorrected sequence divergence comparisons between sister species level were distributed into two different ranges, one closer to 1% (0.5-1.5%) and the other closer to 0% (0-0.5%). The latter divergences were particularly abundant among sister species comparisons within Maio, Boa Vista and Sal. Several mt genomes of different species were almost identical (<0.05%) in sequence including (1) *Africonus delanoyae* and *Africonus luquei*, (2) *Africonus fuscoflavus*, *Africonus* cf. *delanoyae*, and *Africonus messiasi*, (3) *Africonus irregularis* (#1128) and *Africonus cabraloi*, (4) *Africonus verdensis* and *Africonus nelsontiagoi*, and (5) *Africonus gonsaloi* and *Africonus* cf. *gonsaloi*.

### Evolution of radular types

The different lineages within *Africonus* exhibit distinct radular types (Fig. 3). Most lineages and species showed the “robust” type, which is of medium relative size, with a short, pointed barb and a basal spur (see Additional file 1). The anterior section of the tooth is equal or slightly shorter than the posterior section, and the blade covers most of the anterior section (80% – 85%). There are usually 19 to 30 denticles in the serration, arranged in one row (occasionally two). Several species within lineage IV (*Africonus delanoyae*, *Africonus luquei*, *Africonus swinneni*, *Africonus fuscoflavus*, *Africonus messiasi*, *Africonus silviae* and *Africonus* cf. *delanoyae* from Boa Vista island, and *Africonus fantasmalis* from Maio island) exhibited radular teeth of the “elongated” type, similar to the “robust” type but characterized by an anterior section which is longer than the posterior section, a blade covering 40 to 50% of the anterior section, and more numerous denticles in the serration (usually more than 30) often arranged in two rows. Several species (*Africonus borgesi*, *Africonus josephinae* and *Africonus marckeppensi* in lineage I, *Africonus navarroi* in lineage II), all species in lineage III, plus *Africonus vulcanus*, *Africonus miguelfiadeiroi* and *Africonus* cf. *miguelfiadeiroi* in lineage IV) displayed radular tooth of the “broad” type, which is characterized by a medium-sized (Shell Length/Tooth Length = 32-45) and very broad radular tooth (Shell Length/Anterior section Width = 7-12), with an anterior section which is shorter than the posterior section (Tooth Length/Anterior section Length = 2.1-2.9), a blade covering most of the anterior section, and with a variable number of denticles (8 to 30) in the serration arranged in two or more rows. The radular morphology of *Africonus felitae* may represent a special case with a small relative size (Shell Length/Tooth Length = 63-67), narrow (Tooth Length/Anterior section Width = 20-23), the anterior section shorter than the posterior section (Tooth Length/Anterior section Length = 2.2-2.4), and characterized by the total absence of denticles in the serration. The base of this tooth is relatively large and broad.

The species of *Kalloconus* and *Trovaoconus* exhibit essentially two kinds of radular morphologies (Additional file 1). The teeth in *K. pulcher*, and also in *Trovaoconus trochulus*, *T. pseudonivifer* and *Trovaoconus atlanticoselvagem* are narrow and elongated; the blade is moderately short being about one third to almost one-half the length of the anterior section of the tooth, which is distinctly longer than the posterior section of the tooth. There are many denticles (25 to 45 or more) in the long serration, arranged usually in multiple rows with a major row flanked by numerous smaller serrations. In the case of *T. venulatus*, *Trovaoconus ateralbus*, and *Trovaoconus* cf. *ateralbus* the teeth are broader, and the anterior and posterior sections are almost equal in length. There are 16 to 33 denticles in the serration, often coarse and hook-shaped in the middle portion, arranged initially in one row becoming two rows below.

### Dating of major cladogenetic events

**Fig. 4:**
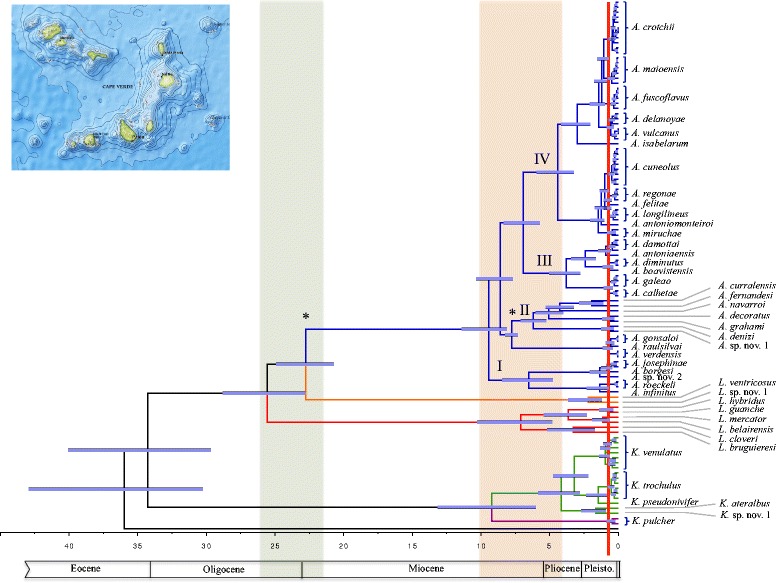
Chronogram based on mt genomes (concatenated protein coding plus rRNA genes analyzed at the nucleotide level) and using the fixed topology of the ML tree shown in Figs. 2 and 3. Lineages are colored as in Fig. 2. Synonymizations proposed (see text) are taken into account. A Bayesian uncorrelated relaxed lognormal clock with geographic-based calibration priors (denoted by asterisks) was used in BEAST. Horizontal bars represent 95% credible intervals for time estimates; dates are in millions of years. Geological periods are indicated. Brown and orange bands indicate main divergence periods around the Oligocene-Miocene and Miocene-Pliocene transitions, respectively. A red line indicates the threshold for species delimitation. The bathymetry of Cabo Verde archipelago is shown in an inset

Major cladogenetic events within the reconstructed phylogeny were dated using an uncorrelated relaxed molecular clock model, which was calibrated using the age of Sal (28 mya; the oldest island of the archipelago) for the node separating *Africonus* from its sister group, *L. ventricosus*, and the age of the origins of São Vicente, Santo Antão, and Santa Luzia (7.5 mya) for the node splitting the lineage including the endemics to these islands from its sister group lineage including endemics to Maio and Santiago islands [24]. The first divergence event involving *Kalloconus* + *Trovaoconus* versus (paraphyletic) *Lautoconus* + *Africonus* was dated at 34 mya (Fig. 4; note that genera and species labels in the chronogram take into account proposed synonymizations, see Discussion). The divergence between the clade containing cones endemic to Senegal (+ *L. guanche*) and the clade including *L. ventricosus* plus *Africonus* was dated at 26 mya. The split between the latter two lineages was dated at 23 mya. The diversification of the crown group of *Africonus* into its four main lineages (I-IV) was estimated to have occurred between 9.4 - 6.9 mya (Fig. 4). The separation of *Kalloconus* and *Trovaoconus* was dated 9 mya and the diversification of the crown group of *Trovaoconus* was established at 4 mya (Fig. 4).

### Diversification rates through time

**Fig. 5:**
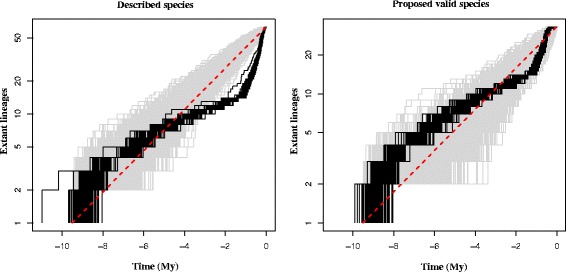
Logarithmic lineage through time (LTT) plots of described (**a**) and proposed valid (**b**) species. The red bar indicates the pure Yule process of speciation, the grey shadow shows 1000 simulated trees and the black lines represent 100 trees randomly chosen among the trees generated by BEAST

Variations in the diversification rates through time were estimated for *Africonus* and the hypothesis of a radiation during the evolutionary history of the clade was tested. The gamma-statistic, which measures departures from a constant rate of diversification, had values of 7.19 (*p* < 0.05) and 3.12 (p < 0.05) when considering the currently named (based on phenotypic traits) or only the here-proposed (considering genetic evidence) species for the genus, respectively. In both cases, the hypothesis of a radiation is accepted. According to the lineage through time plots (Fig. 5), the initial rate of increase in the number of species slowed down between six and one and a half million years ago regardless of the species delimitation hypothesis tested. Afterwards, the diversification rate accelerated considerably, and the increase in number of species either continued or abandoned a normal Yule process of speciation when considering the species delimitation hypothesis here proposed or the currently number of named species, respectively.

## Discussion

Cone snails are marine gastropods well known to evolutionary biologists due to their extraordinary species and ecological diversity [25], but also to molecular biologists and pharmacologists due to their sophisticated venom cocktails [26], as well as to amateur naturalists due to their brightly colored and highly appreciated shells [27]. Therefore, they are the subject of intensive research across disciplines and additionally have received wide attention from the general public. There are more than 800 described species and this number increases steadily every year [28]. Thus far, species description and identification of cones heavily relies on shell form, color and banding patterns, which may show great variety at local scale leading to important levels of synonymy within the family Conidae [7, 20]. In this regard, species delimitation could greatly improve with the aid of robust molecular phylogenies, which could be used in addition as framework to uncover the evolutionary patterns and processes underlying the diversification of the group. While reconstructing a robust phylogeny for all described cone species worldwide is cumbersome and at present unrealistic within the framework of a single study, it is possible, however, to accomplish a proof-of-concept study in a particular region [23].

We have here reconstructed a molecular phylogeny of cones endemic to Cabo Verde and allied species in the Macaronesian region, continental West Africa, and the Mediterranean region. These cones are particularly interesting from an evolutionary perspective as they have radiated in an oceanic archipelago and constitute a natural experiment to gain insights onto the processes governing diversification and adaptation [29]. Phylogenetic analyses were based on nearly complete mt genomes (only missing the control region and neighboring sequences) and included 105 specimens comprising most of the cone species diversity of the analyzed regions. Probabilistic methods of phylogenetic inference arrived at a robust and highly resolved phylogeny (virtually all nodes received high statistical support, which in most cases was maximal). To our knowledge, this is the first wide application of mt genomes to the resolution of a phylogeny within mollusks (but see [30, 31] for comparable examples in fish and insects, respectively). Previous studies in gastropods were restricted in the number of taxa analyzed (e.g., [22] for the family Conidae) but here we were able to achieve a lineage representation of the reference group (in this case, Cabo Verde cones) only previously attained by studies using few concatenated partial gene sequences (see e.g., [25] for the family Conidae or [1] for the cones of Cabo Verde). Previous phylogenetic studies using complete mt genomes have demonstrated that the level of resolution of these molecular markers is compromised above the superfamily level due to saturation, base composition biases, and among-lineage rate heterogeneity [32, 33]. Here, we show that phylogenetic performance of mt genomes achieves best results when analyzing closely related genera (and their corresponding species). Moreover, results were particularly promising taking into account that *Africonus* diversity in Cabo Verde was originated through radiation processes, which normally lead to relatively short tree nodes (often difficult to disentangle).

A thorough sampling of closely-related outgroup taxa allowed us to tackle key questions on the origin of the cones endemic to Cabo Verde and on their closest living sister groups. As previously reported, there are two independent origins of Cabo Verde cones, leading to the genera *Africonus* and *Trovaoconus*, respectively [1]. The closest living sister group of *Africonus* is *L. ventricosus* from the Mediterranean Sea and neighboring Atlantic Ocean. Therefore, the origin of this clade is clearly Macaronesian/Mediterranean and these cones are only distantly related to the geographically closer cones endemic to Senegal. These latter cones were ascribed to the genus *Lautoconus* (as was the case of *L. guanche* from Canary islands, deeply nested within the clade of Senegal cones; [23]). However, the closest sister group relationship of *Africonus* and *L. ventricosus* requires formal description of a different genus for Senegal cones (plus *L. guanche*), which will be done elsewhere. We could not include any representative of cones endemic to Angola (genus *Varioconus*) but a recent phylogeny based on partial *cox1* gene sequences recovered all these cones (including *Varioconus jourdani* from Saint Helena Island) as a monophyletic group sister to Senegal cones [34]. Alternatively, all previously mentioned genera could be merged into genus *Lautoconus* [35]. However, the relatively high levels of sequence divergence (using the sequence divergence of genus *Chelyconus* as reference) and the restricted (endemic) distribution of the clades fit better with the former taxonomic proposal. The closest living sister group of *Trovaoconus* is genus *Kalloconus* from West Africa. Therefore, the origin of this clade is clearly related to neighboring regions of the continent. Actually, the sequence divergence between *Trovaoconus* and *Kalloconus* is much lower than that estimated between *Lautoconus* and *Africonus*. This observation argues against maintaining the generic status of *Trovaoconus*, and supports the inclusion of their species within genus *Kalloconus*, as some authors have proposed [35]. Hence, *Kalloconus* would be a genus that is present throughout the coast of West Africa from Morocco to Angola as well as in Canary Islands and Cabo Verde.

Altogether, the two main clades in the reconstructed phylogeny show very distinct patterns of distribution. One clade includes a single genus with widespread distribution in Macaronesia and West Africa whereas the other clade, which occupies the same geographical regions, is divided into several valid genera (*Africonus*, *Lautoconus*, *Varioconus*, Gen. nov. for Senegal endemics). These distinct patterns could be explained partly taking into account differences in larval dispersal capabilities between the two clades [1]. According to the phylogeny, the ancestor of the *Kalloconus* clade was inferred to have planktotrophic larvae, capable of long dispersals whereas the ancestor of the other clade would have non-planktotrophic larvae, and thus a limited dispersal capability leading to restricted gene flow and higher rates of diversification [36, 37]. Interestingly, the ancestor of *Kalloconus* species endemic to Cabo Verde (former *Trovaoconus* species) lost planktotrophy, which is a common evolutionary pattern in insular species [38].

According to the reconstructed phylogeny, cones belonging to genus *Africonus* are divided into four main lineages (I-IV; with each further subdivided into two distinct clades). Species endemic to Maio and Boa Vista are found in all four lineages whereas species endemic to Sal form a clade within lineage IV, species from the westernmost islands (Santo Antão, São Vicente and Santa Luzia) form a clade within lineage II, and the single species from Santiago is recovered within lineage II. Unfortunately, we could not sample specimens of *Africonus furnae* from Brava and *Africonus kersteni* from São Nicolau, and cannot determine whether they could be ascribed to any of the above-mentioned four lineages or form their own independent lineages. The single origin of cones endemic to Sal, Santiago, and westernmost islands could be explained by the deep slopes separating these islands whereas the multiple origins of the cones found in Maio and Boa Vista could be associated to the relatively shallow seamount (Baixo João Valente) connecting both islands [1]. These differences in bathymetry in connection with past eustatic sea level changes could be determinant in preventing or promoting dispersal in *Africonus* species, whose larvae are all non-planktotrophic.

Diversification events among main lineages were concentrated in three major periods. The first one, around the Oligocene-Miocene boundary (23 mya), includes the divergence of cones endemic to Senegal (and Angola) from their sister clade, and the posterior separation within this sister clade of cones endemic to Cabo Verde and those endemic to the Mediterranean Sea and neighboring Atlantic Ocean. During Oligocene-Miocene transition, there was a global cooling event [39, 40], the ice sheet of Antarctica greatly expanded, and a sea level drop of ~50 m occurred [41]. The second period corresponds to a sustained global cooling in the Late Miocene starting 12 mya [42] that produced an eustatic sea level drop between −10 and −30 m from 6.26 to 5.50 Mya [43] and culminated with the Messinian Salinity crisis and the desiccation of the Mediterranean Sea at the end of the Miocene from 5.96 to 5.33 Mya [44]. During this period, the divergence of the main lineages within *Africonus* (I-IV), the cones endemic to Senegal, and *Kalloconus* occurred. Finally, a burst of speciation events is inferred during the Pleistocene when another cooling period characterized by extreme climate oscillations and drastic eustatic sea level changes concurring with glacial-interglacial periods [45]. Global cooling has been recently proposed to be a driver of diversification of marine species [46] in agreement with our results. The reconstructed phylogeny, the chronogram, and the current geographical distribution of the species altogether support that allopatry is the main mode of speciation for cone snails with non-planktotrophic larvae, as previously suggested [1]. The complex geology of the island of Boa Vista with several eruptions at >16, 15-12.5, and 9.5-4.5 mya [47], involving different parts of the island may have also contributed to creating additional niches along the coast and could explain that this island harbors the highest number of endemic cones.

The reconstructed phylogeny also allows inferring the evolution of the radula in the studied lineages [8, 48]. All analyzed ingroup taxa are vermivorous [8]. Studies documenting potential specialization of the vermivore radular type to prey on specific worm species are scarce and restricted thus far to cone species preying on amphinomids [49]. Here, we show that most *Africonus* species show a “robust” radular type, which is shared also with *L. ventricosus* and a lineage of Senegal cones represented by *Lautoconus cloveri* and *Lautoconus bruguieresi* in the phylogeny [23]. Therefore, the common ancestor of cones endemic to Senegal (plus *L. guanche*), *Africonus*, and *L. ventricosus* was inferred to have a “robust” type radula. The “elongated” type of radular tooth, which was found in several species within lineage IV of *Africonus*, also appears in a lineage of Senegal cones that is represented by *Lautoconus hybridus* and *L. guanche* in the phylogeny [23]. The radular tooth of *A. felitae* resembles the “small” type observed in a lineage of Senegal cones represented by *Lautoconus reticulatus* and *Lautoconus belairensis* in the phylogeny [23]. The “broad” type of radular tooth that appeared independently in several lineages of *Africonus* has not been observed in any cone from Senegal. While shifts in radular type could be correlated with early cladogenesis in cones endemic to Senegal [23], the evolution of different types of radular tooth within *Africonus* was restricted to few specific cases. Thus, future studies are needed to determine whether in such cases there has been a dietary shift to prey on specific worms. The radula teeth identified in *Kalloconus* resemble the types “elongated” and “robust” observed within *Lautoconus* and *Africonus*, although are clearly distinct. This might indicate instances of convergence, and that only a discrete number of different main types of radula could be found in a given clade.

During the last few years, the number of new cone species described from Cabo Verde has increased at an astonishing rate (e.g.*,* [12]). These new species are identified based on differences (often subtle) in shell shape and color, and their status needs to be contrasted with genetic data to uncover cases of local phenotypic variation within species due to either genetic polymorphism or phenotypic plasticity that may be producing overestimations of the number of species in the group [21]. In addition, genetic data could help identify cases of phenotypic convergence due to adaptation of genetically distinct populations (ecotypes) or species (sibling or cryptic) to similar environments [50–52], also affecting the total number of valid species. Comparative analyses of pairwise uncorrected sequence divergences taking into account the reconstructed phylogeny showed that some described species shared almost identical mt genomes with levels of sequence divergence normally considered to be associated to genetic variation at the population level. Clades comprising these sets of closely related sequences indicate that an uncorrected sequence divergence threshold around 1% could be associated to the species status. This threshold lies well within the so-called grey zone of speciation between 0.5-2% [53]. Of course, these results need to be further confirmed with genomic nuclear data that discard potential events of incomplete lineage sorting and hybridization [54]. In addition, the present study could be further improved in the future by increasing the number of individuals analyzed per original species. Importantly, the comparative analyses on variation of diversification rates through time support the here proposed hypothesis of species delimitation as it concurs with a Yule process of speciation whereas the number of currently named species clearly exceed expectations and would imply an extraordinary recent acceleration of speciation rates.

Our study confirms the diversity of cone endemic to Cabo Verde but significantly reduces the number of valid species. Applying the threshold in a conservative manner (i.e., maintaining described species as valid in case of doubt due to closeness to the threshold) to cones endemic to Cabo Verde would reduce the number of valid species within the sampled *Africonus* from 65 to 32 (see Table 1 and Fig. 4). The proposed nomenclatural changes follow standard ICZN recommendations maintaining the most senior (oldest) name. Among the species not sampled, two correspond to São Nicolau and Brava islands, four of them are from the islands of São Vicente and Santa Luzía, and most likely represent valid species (*Africonus bellulus*, *Africonus lugubris*, *Africonus saragasae*, *Africonus santaluziensis*, *A. kersteni* and *A. furnae*) given the relative high sequence divergences found among species endemic to these islands. The 19 remaining ones were recently described, mostly from Boa Vista, and are expected to fall in most cases into some of the clades already discussed in the present work, and therefore may correspond to morphs of other described species. A direct consequence of synonymization is that some previously described species of rather restricted distribution are merged as populations into the new species, which considerably increase their range of distribution (Additional file 1). For instance, *A. crotchii*, which was reported as endemic from Southwest Boa Vista, would be now distributed also in the whole north half of the island. This increase in range of distribution of several species has important effects on their IUCN conservation status [3]. In the case of *Kalloconus*, some morphotypes attributed to *Kalloconus pseudonivifer* are now assigned to *Kalloconus trochulus*, and *Kalloconus atlanticoselvagem* is synonymized with *K. trochulus*. Our specimen of *Kalloconus* cf. *byssinus* is from North Senegal and has little sequence divergence compared to *Kalloconus pulcher*. In this case, it would be important to study *K. byssinus* from Mauritania or Morocco before considering synonymization. In the opposite direction, there are three clear instances of morphological convergence and thus, of the existence of cryptic species. Those are the cases of *Africonus josephinae* from Maio, *Africonus* cf. *miruchae* from São Vicente, and *Kalloconus* cf. *ateralbus* from Sal, which will be described as new species in due course.

## Conclusions

We reconstructed a robust phylogeny based on mitochondrial genomes of cone snails endemic to Cabo Verde, which provides the necessary framework for future evolutionary studies focused on this radiation. The double origin of Cabo Verde endemic cones was supported. The ancestor of *Africonus* separated from L. ventricosus during the Oligocene-Miocene boundary (about 23 mya) and diversified into four main lineages (I to IV) in the Late Miocene (about 9.4-6.9 mya). The divergence of the ancestor of *Kalloconus* endemic to Cabo Verde from those inhabiting mainland occurred also in the Late Miocene whereas its diversification into three main lineages was dated in the Pliocene (4 mya). Main cladogenetic events within cones endemic to Cabo Verde coincide with global cooling periods, which were characterized by radical climate oscillations and eustatic sea level changes. Recurrent cycles of island connection/ disconnection likely favored speciation in allopatry in these cones, which lack a pelagic larval stage, and thus have limited dispersal capacity. Direct development evolved in the ancestor of *Kalloconus* endemic to Cabo Verde, likely associated to the colonization of the archipelago by a cone with a planktotrophic larval stage. However, in the case of *Africonus*, the ancestor that arrived to Cabo Verde was already non-planktotrophic as the corresponding independent evolutionary shift to direct development predated the separation of cones endemic to Senegal (and Canary Islands) from *L. ventricosus* plus *Africonus*. Radular types were modified during the diversification of *Africonus* from an ancestral “robust” type, although correlation with diet specializations await better knowledge of the specific worm species preyed by the different species of cones. Sequence divergence comparisons and reconstructed phylogenies supported the diversity of cone species endemic to Cabo Verde but significantly reduced its number, which was likely overestimated in the past due to important homoplasy in shell morphology, the, thus far, main discriminant character used for species description and identification.

## Methods

### Samples and DNA extraction

The complete list of specimens analyzed in this study corresponding to different populations and species of *Africonus* and *Trovaoconus* from Cabo Verde is shown in Table 1, as well as details on the respective sampling localities and museum vouchers. As outgroup taxa, we also sampled and analyzed one specimen of *L. ventricosus* from Formentera Island (Spain). Specimens were collected by snorkel at 1-3 m depth, or picked by hand at low tide. All samples were stored in 100% ethanol. The initial species identification (see corresponding column in Table 1) was based on comparison with type material (mostly deposited in the MNCN) or consulting the original publications. Total DNA was isolated from 5 to 10 mg of foot tissue following a standard phenol-chloroform extraction [55].

### Radular tooth preparation

The radular sac was dissected from the main body and soft parts were digested in concentrated aqueous potassium hydroxide for 24 h. The resulting mixture was then placed in a petri dish and examined with a binocular microscope. The entire radula was removed with fine tweezers and rinsed with distilled water, then mounted on a slide using Aquatex (Merck, Germany) mounting medium, and observed under a compound microscope. Photographs were taken with a charge-coupled device (CCD) camera attached to the microscope. Terminology for radular morphology follows [8], with abbreviations following [48]. Names of radular types follow [23].

### PCR amplification and sequencing

Near-complete (without the control region) mt genomes were amplified through a combination of standard and long PCRs using the primers and following the protocols of [22]. Standard-PCR products were sequenced using Sanger technology. Long-PCR products were subjected to next-generation sequencing. Briefly, PCR amplified fragments from the same mt genome were pooled together in equimolar concentrations. For each cone mt genome a separate indexed library was constructed using the NEXTERA XT DNA library prep kit (Illumina, San Diego, CA, USA). The average size of the Nextera libraries varied between 307 and 345 bp. Libraries were pooled and run in an Illumina MiSeq platform (v.2 chemistry; 2 × 150 paired-end) at Sistemas Genómicos (Valencia, Spain).

### Genome assembly and annotation

The reads corresponding to each mt genome were sorted using the corresponding library indices, and read assembly was performed in the TRUFA webserver [56]. Briefly, adapters were removed using SeqPrep [57], quality of the reads was checked using FastQC v.0.10.1 [58], and raw sequences were trimmed and filtered out according to their quality scores using PRINSEQ v.0.20.3 [59]. Filtered reads were used for de novo assembly of each mt genome using default settings (minimum contig length: 200; sequence identity threshold: 0.95) of Trinity r2012-06-08 [60] in TRUFA, and only retaining contigs with a minimum length of 3 kb. These contigs were used as starting point to assemble the mt genomes using Geneious® 8.0.3. First, the (raw) reads with a minimum identity of 99% were mapped against the contigs to correct possible sequence errors. Then, successive mapping iterations using a 100% identity as threshold were performed to elongate the contigs.

The mt genomes were annotated with the option “Annotate from Database” in Geneious® 8.0.3, using published mt genomes of Conidae as references. Annotations of the 13mt protein-coding genes were refined manually identifying the corresponding open reading frames using the invertebrate mitochondrial code. The transfer RNA (tRNA) genes were further identified with tRNAscan-SE 1.21 [61], which infer cloverleaf secondary structures (with a few exceptions that were determined manually). The ribosomal RNA (rRNA) genes were identified by sequence comparison with other Conidae mt genomes [22], and assumed to extend to the boundaries of adjacent genes [62]. GenBank accession numbers of each mt genome are provided in Table 1.

### Sequence alignment and phylogenetic analyses

The newly sequenced mt genomes were aligned with the mt genomes of *A. borgesi*, *Africonus infinitus*, *Africonus miruchae*, *T. ateralbus*, *T. pseudonivifer*, *T. venulatus*, and *C. ermineus* from Cabo Verde, *L. hybridus, L. mercator, L. belairensis, L. cloveri, L. bruguieresi*, *K. pulcher*, and *K.* cf. *byssinus* from Senegal, *L. guanche* from Canary Islands, and *L. ventricosus* from Portugal, which were downloaded from GenBank (Table 1). A sequence data set was constructed concatenating the nucleotide sequences of the 13 mt protein-coding and two rRNA genes. The deduced amino acid sequences of the 13 mt protein-coding genes were aligned separately and used to guide the alignment of the corresponding nucleotide sequences with Translator X [63]. Nucleotide sequences of the mt rRNA genes were aligned separately using MAFFT v7 [64] with default parameters. Ambiguously aligned positions were removed using Gblocks, v.0.91b [65] with the following settings: minimum sequence for flanking positions: 85%; maximum contiguous non-conserved positions: 8; minimum block length: 10; gaps in final blocks: no. Finally, the different single alignments were concatenated using Geneious® 8.0.3. Sequences where format converted for further analyses using the ALTER webserver [66]. The concatenated alignment is available at http://purl.org/phylo/treebase/phylows/study/TB2:S21557.

Phylogenetic relationships were inferred using maximum likelihood (ML, [67]) and Bayesian inference (BI, [68]). For ML, we used RAxML v8.1.16 [69] with the rapid hill-climbing algorithm and 10,000 bootstrap pseudoreplicates (BP). BI analyses were conducted with MrBayes v3.1.2 [70], running four simultaneous Markov chains for 10 million generation, sampling every 1000 generations, and discarding the first 25% generations as burn-in (as judged by plots of ML scores and low SD of split frequencies) to prevent sampling before reaching stationarity. Two independent Bayesian inference runs were performed to increase the chance of adequate mixing of the Markov chains and to increase the chance of detecting failure to converge, as determined using Tracer v1.6 [71]. The effective sample size (ESS) of all parameters was checked to be above 200. Node support was assessed based on Bayesian Posterior Probabilities (BPP). A node was considered highly supported with BP and BPP values above 70% and 0.95, respectively. The ML and BI phylogenetic trees are available at http://purl.org/phylo/treebase/phylows/study/TB2:S21557.

The best partition schemes and best-fit models of substitution for the data set were identified using PartitionFinder2 [72] with the Akaike information criterion [73]. For the protein-coding genes, the partitions tested were: all genes grouped; all genes separated (except *atp6*-*atp8* and *nad4*-*nad4L*); and genes grouped by subunits (*atp*, *cob, cox*, and *nad*). In addition, these three partitions schemes were tested taking into account separately the three codon positions. The rRNA genes were tested with two different schemes, genes separated or combined.

### Estimation of divergence times

The program BEAST v.1.8.0 [74] was used to perform a Bayesian estimation of divergence times. An uncorrelated relaxed molecular clock was used to infer branch lengths and nodal ages. The tree topology was fixed using the one recovered by the ML analysis. For the clock model, the lognormal relaxed-clock model was selected, which allows rates to vary among branches without any a priori assumption of autocorrelation between adjacent branches. For the tree prior, a Yule process of speciation was employed. Concatenated protein coding plus rRNA genes were analyzed at the nucleotide level. The partitions and models selected by PartitionFinder2 were applied (see results). The final Markov chain was run twice for 100 million generations, sampling every 10,000 generations, and the first 1000 trees were discarded as part of the burn-in process, according to the convergence of chains checked with Tracer v.1.5. [71]. The ESS of all parameters was above 200.

Despite the fact that there are many fossils of Conidae, it is difficult in many instances to be certain about species identifications given the important levels of homoplasy in shell shape [75]. Hence, although there are fossils attributed to *L. ventricosus* [76] and *L. mercator* [77], which could be applied to the reconstructed phylogeny, we opted to calibrate the clock using biogeographical events (i.e., the age of the islands of Cabo Verde). We run a preliminary analysis in which the posterior distribution of the estimated divergence times was obtained by specifying one calibration point as prior for the divergence time of the split between *L. ventricosus* and the genus *Africonus*. This genus is endemic to Cabo Verde, and we used the age of formation of the oldest island, Sal (28 Mya; [24]), as biogeographical calibration point. We applied a log-normal distribution as the prior model for the calibration and enforced the median divergence time to equal 25 (s.d. = 0.05, offset = 0.7). According to the results of the preliminary analysis, we found that only in the case of São Vicente, Santo Antão, and Santa Luzia, the early divergence of living cone endemic lineages followed the origin of the corresponding island, and therefore, we used a second calibration point corresponding to the origin of these islands about 7.5 mya [24]. We applied a log-normal distribution as the prior model for the calibration and enforced the median divergence time to equal 7.5 (s.d. = 0.03, offset = 0). The BEAST tree is available at http://purl.org/phylo/treebase/phylows/study/TB2:S21557.

### Diversification rate through time

The chronogram was used to determine diversification rate through time of genus *Africonus* under alternative (phenotypic versus genetic) species delimitation hypotheses. A lineage through time (LTT) plot analysis was conducted using the APE 4.1 R package [78]. A random sample of 100 trees was selected and mapped over a simulation of 1000 trees following a Yule process of speciation (net diversification rate = 0.4). The phytools R package [79] was used to calculate the Gamma-Statistic [80].

## Additional file

Additional file 1: Maps showing sampling localities; diversity of Kalloconus radular teeth. (ZIP 3996 kb)
