# Supplementary material for: Phylogenetic relationships of cone snails endemic to Cabo Verde based on mitochondrial genomes
Source: BMC Evol Biol. 2017 Nov 25;17:231. doi: 10.1186/s12862-017-1069-x (PMC5702168; doi:10.1186/s12862-017-1069-x)
Supplement: Additional file 1: — Maps showing sampling localities; diversity of Kalloconus radular teeth. (ZIP 3996 kb) [file 12862_2017_1069_MOESM1_ESM.zip › appendix map.pdf]

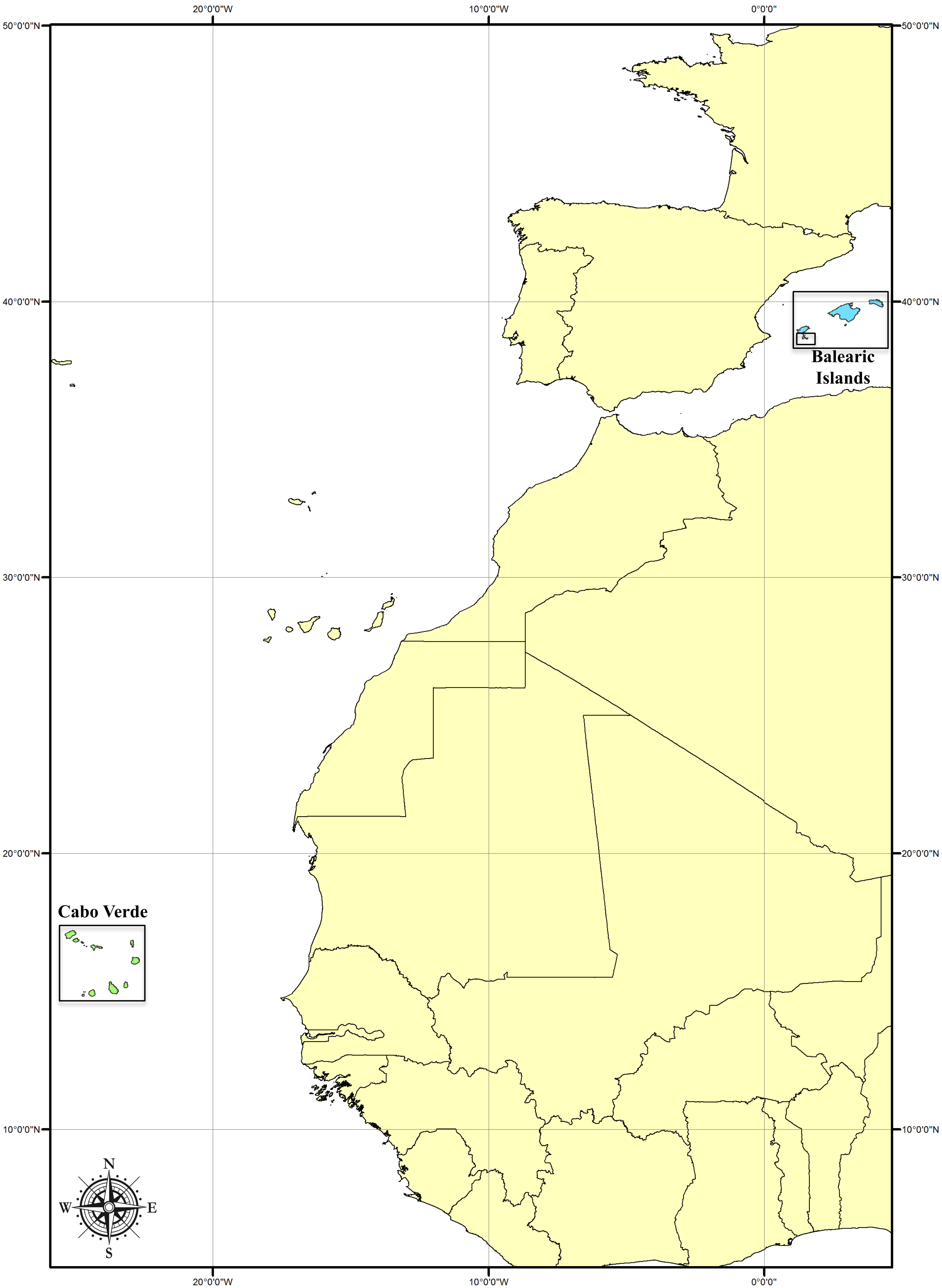

20°0'0"W
10°0'0"W
0°0'0"
50°0'0"N
40°0'0"N
30°0'0"N
20°0'0"N
10°0'0"N
Balearic Islands
Cabo Verde
N
S
E
W

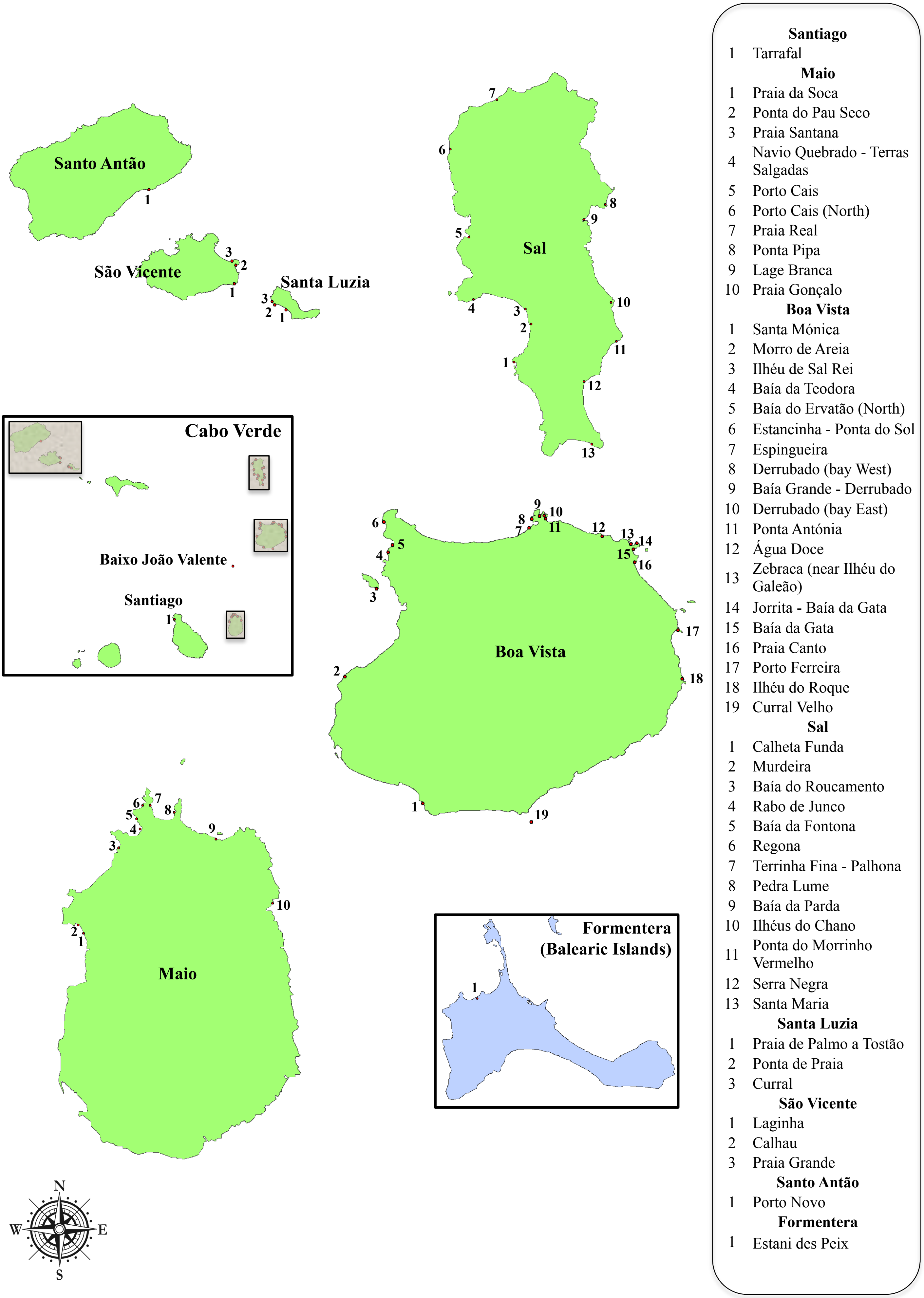
Santo Antão
São Vicente
Santa Luzia
Sal
Boa Vista
Maio
Cabo Verde
Baixo João Valente
Santiago
Formentera
(Balearic Islands)
N
E
S
W
Santiago
1 Tarrafal
Maio
1 Praia da Soca
2 Ponta do Pau Seco
3 Praia Santana
4 Navio Quebrado - Terras Salgadas
5 Porto Cais
6 Porto Cais (North)
7 Praia Real
8 Ponta Pipa
9 Lage Branca
10 Praia Gonçalo
Boa Vista
1 Santa Mónica
2 Morro de Areia
3 Ilhéu de Sal Rei
4 Baía da Teodora
5 Baía do Ervatão (North)
6 Estancinha - Ponta do Sol
7 Espingueira
8 Derrubado (bay West)
9 Baía Grande - Derrubado
10 Derrubado (bay East)
11 Ponta Antónia
12 Água Doce
13 Zebraca (near Ilhéu do Galeão)
14 Jorrita - Baía da Gata
15 Baía da Gata
16 Praia Canto
17 Porto Ferreira
18 Ilhéu do Roque
19 Curral Velho
Sal
1 Calheta Funda
2 Murdeira
3 Baía do Roucamento
4 Rabo de Junco
5 Baía da Fontona
6 Regona
7 Terrinha Fina - Palhona
8 Pedra Lume
9 Baía da Parda
10 Ilhéus do Chano
11 Ponta do Morrinho Vermelho
12 Serra Negra
13 Santa Maria
Santa Luzia
1 Praia de Palmo a Tostão
2 Ponta de Praia
3 Curral
São Vicente
1 Laginha
2 Calhau
3 Praia Grande
Santo Antão
1 Porto Novo
Formentera
1 Estani des Peix
