# Supplementary material for: Phylogenetic relationships of cone snails endemic to Cabo Verde based on mitochondrial genomes
Source: BMC Evol Biol. 2017 Nov 25;17:231. doi: 10.1186/s12862-017-1069-x (PMC5702168; doi:10.1186/s12862-017-1069-x)
Supplement: Additional file 1: — Maps showing sampling localities; diversity of Kalloconus radular teeth. (ZIP 3996 kb) [file 12862_2017_1069_MOESM1_ESM.zip › appendix radula.pdf]

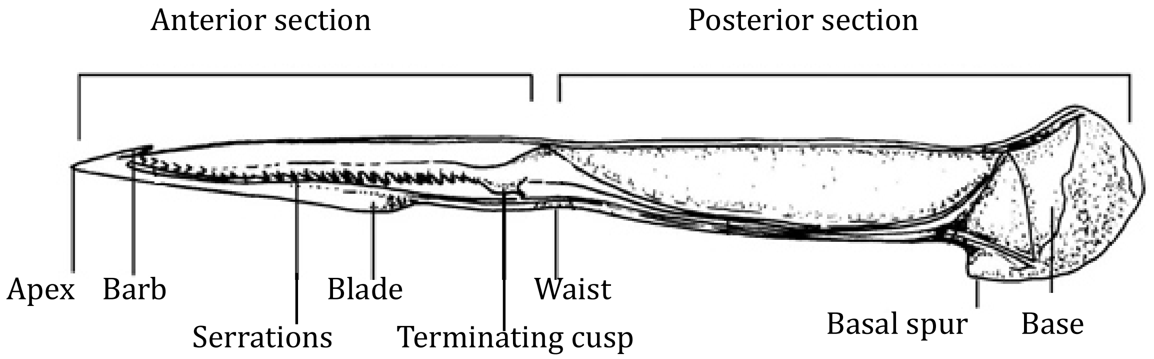

Radular teeth of *Kalloconus*

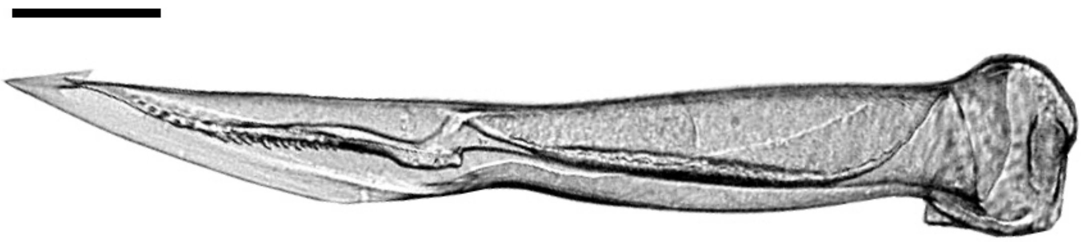

Kalloconus ateralbus 0601 Calheta Funda, Sal, Cabo Verde

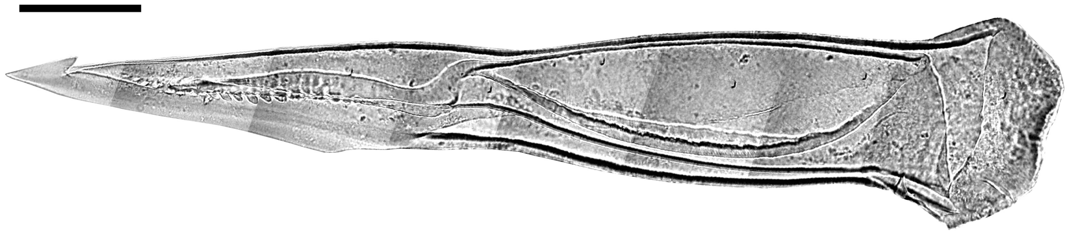

Kalloconus cf. *ateralbus* 0616 Serra Negra, Sal, Cabo Verde

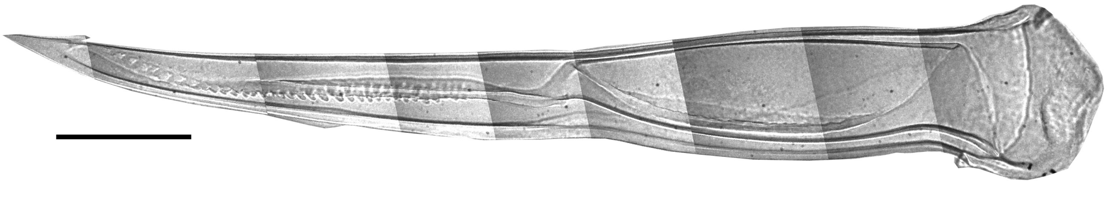

Kalloconus pseudonivifer 0420 Praia Canto, Boa Vista, Cabo Verde

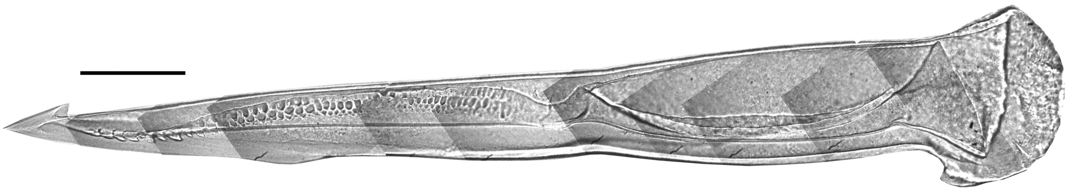

Kalloconus trochulus 0495 Ervatão, Boa Vista, Cabo Verde

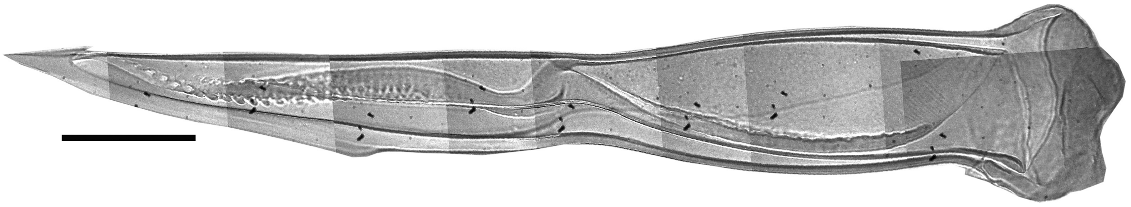

Kalloconus venulatus 0550 Ilhéu de Sal Rei, Boa Vista, Cabo Verde

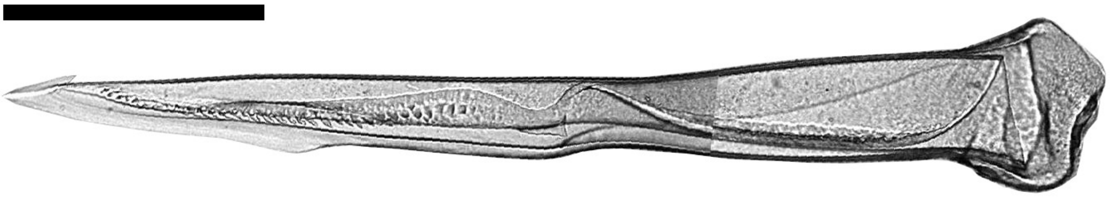

Kalloconus cf. *byssinus* 0601 North Senegal

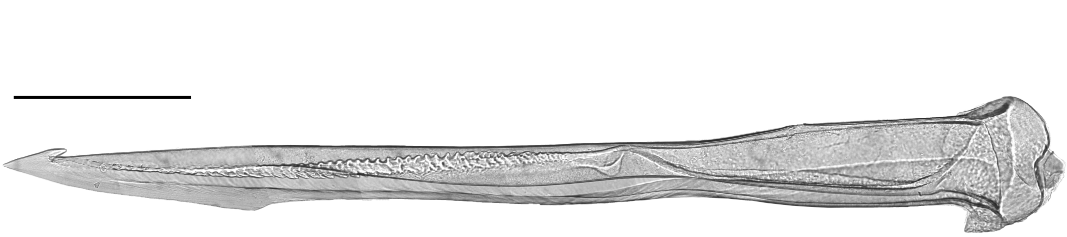

Kalloconus pulcher 1316 Joal Fadiouth, Senegal
